# The role of programmed cell death 1 in autoimmune diseases: mechanisms and therapeutic implications

**DOI:** 10.3389/fimmu.2026.1707084

**Published:** 2026-02-24

**Authors:** Zhenyu Liu, Zipeng Hu, Huilin Lao, Lemin Chen, Yue Wen, Yuqiang Liang, Chong Zhang, Jiang Wu, Xianliang Hou

**Affiliations:** 1 Laboratory Center, Guangxi Key Laboratory of Metabolic Reprogramming and Intelligent Medical Engineering for Chronic Diseases, the Second Affiliated Hospital of Guilin Medical University, Guilin, China; 2 Department of Nuclear Medicine, Jinling Hospital, Affiliated Hospital of Medical School, Nanjing University, Nanjing, China; 3 Department of Central Laboratory, Shenzhen Hospital, Beijing University of Chinese Medicine, Shenzhen, Guangdong, China

**Keywords:** autoimmune diseases, immune tolerance, immunotherapy, PD-1, PD-L1

## Abstract

Autoimmune diseases are complex disorders caused by the interaction between the immune system and self-antigens, involving genetic, environmental triggers, and other cellular factors. The programmed cell death receptor-1 (PD-1) gene, as a critical factor in immune regulation, has garnered significant attention in the study of autoimmune diseases such as rheumatoid arthritis, systemic lupus erythematosus, and ankylosing spondylitis. Although the pathogenesis of these diseases varies, they all manifest as a breakdown in immune tolerance and an imbalance in immune homeostasis. Research has shown that in the development of autoimmune diseases, changes in PD-1 gene expression, its binding with PD-L1 and PD-L2, and signal transduction pathways are often abnormal. These abnormalities may lead to the overactivation of T cells and B cells, resulting in the attack on self-tissues. Consequently, therapeutic strategies targeting the PD-1/PD-L1 signaling pathway hold promising potential. Gene therapy approaches or small-molecule drugs that enhance PD-L1 transcription could strengthen PD-1/PD-L1 binding and restore inhibitory signaling, thereby rebalancing immune responses and improving patients’ quality of life. Additionally, recent studies suggest that targeting Vγ4γδT cells to monitor disease progression and prognosis represents another potential PD-1-based therapeutic strategy. This review focuses on the role of the PD-1 gene in autoimmune diseases, systematically elaborating on the structure, molecular functions, and regulatory mechanisms of PD-1 and its ligands, while providing an in-depth analysis of PD-1’s mechanistic involvement in autoimmune diseases and its therapeutic prospects.

## 1 Introduction

PD-1 and its ligands, as key co-inhibitory molecules, contribute significantly to inhibiting T cell signal transduction, mediating immune tolerance, and maintaining immune homeostasis. The binding of PD-1 with its ligands initiates negative regulatory signals that suppress excessive immune responses thereby preserving immune tolerance and homeostasis. Dysfunction of PD-1 or its ligands can precipitate various autoimmune diseases. Autoimmune diseases are a group of disorders characterized by the immune system’s abnormal attack on self-tissues, with complex pathogenesis involving genetic, environmental, and immune factors (1–4). In recent years, in-depth studies of immune regulation mechanisms have drawn widespread attention to the role of PD-1 as a critical immune checkpoint molecule in autoimmune diseases (5, 6).

## 2 Molecular structures and basic functions of PD-1 and its ligands

Programmed cell death protein 1 (PD-1) is a single-chain transmembrane protein belonging to the CD28/CTLA-4 family and is one of the immune checkpoint molecules. Unlike other CD28 family members, PD-1 exists as a monomer on the cell surface. Located on human chromosome 2q37.3, PD-1 contains five exons and four introns. The PD-1 protein, encoded by exons, includes a signal sequence, an IgV domain, an IgC domain, and an intracellular immunoreceptor tyrosine-based inhibitory motif (ITIM) domain (6). PD-1 primarily regulates immune cell activation and function by binding to its ligands, programmed death-ligand 1 (PD-L1) and programmed death-ligand 2 (PD-L2), thereby inhibiting T cell activation and function, regulating the magnitude and duration of immune responses, and maintaining immune tolerance and balance (7–9).

PD-L1 is a transmembrane protein featuring an extracellular region composed of Ig-V and Ig-C domains, a transmembrane domain, and a short cytoplasmic tail that lacks a canonical signaling motif (10, 11). PD-L1 is expressed on hematopoietic cells such as T cells, B cells, dendritic cells, macrophages, mesenchymal stem cells, and bone marrow-derived mast cells, as well as on non-hematopoietic cells such as vascular endothelial cells, liver non-parenchymal cells, pancreatic islets, and keratinocytes (12). PD-L2, a type I transmembrane protein of the B7 family, also contains Ig-V and Ig-C2 domains (13). Its expression is prominent not only in many cancers but also in immune cells such as macrophages and dendritic cells (14–16). Although PD-L1 is widely expressed (17), PD-1 exhibits a higher binding affinity for PD-L2 than for PD-L1. The detailed molecular architectures and binding interfaces of PD-1 with its ligands have been elucidated by structural studies (e.g., X-ray crystallography), revealing the biophysical basis for their interaction and differential affinity; readers are referred to comprehensive reviews on this topic for visual representations (11, 18, 19).

## 3 Detailed molecular functions of PD-1 and its ligands

The PD-1 gene exerts diverse molecular functions, most prominently in the contexts of tumor immunology and autoimmune diseases. These functions are fundamentally linked through its core mechanism of regulating immune responses via the PD-1/PD-L1 signaling axis. A detailed understanding of this functionality is therefore crucial for developing targeted therapeutic strategies. Single nucleotide polymorphisms (SNPs) in the PD-1 gene may affect the structure and function of the PD-1 protein (20), altering its binding ability with PD-L1 or PD-L2, thereby influencing immune cell activation and function and increasing the risk of autoimmune diseases (21, 22).

### 3.1 Core signaling and its role in immune regulation and tolerance

**Figure 1:**
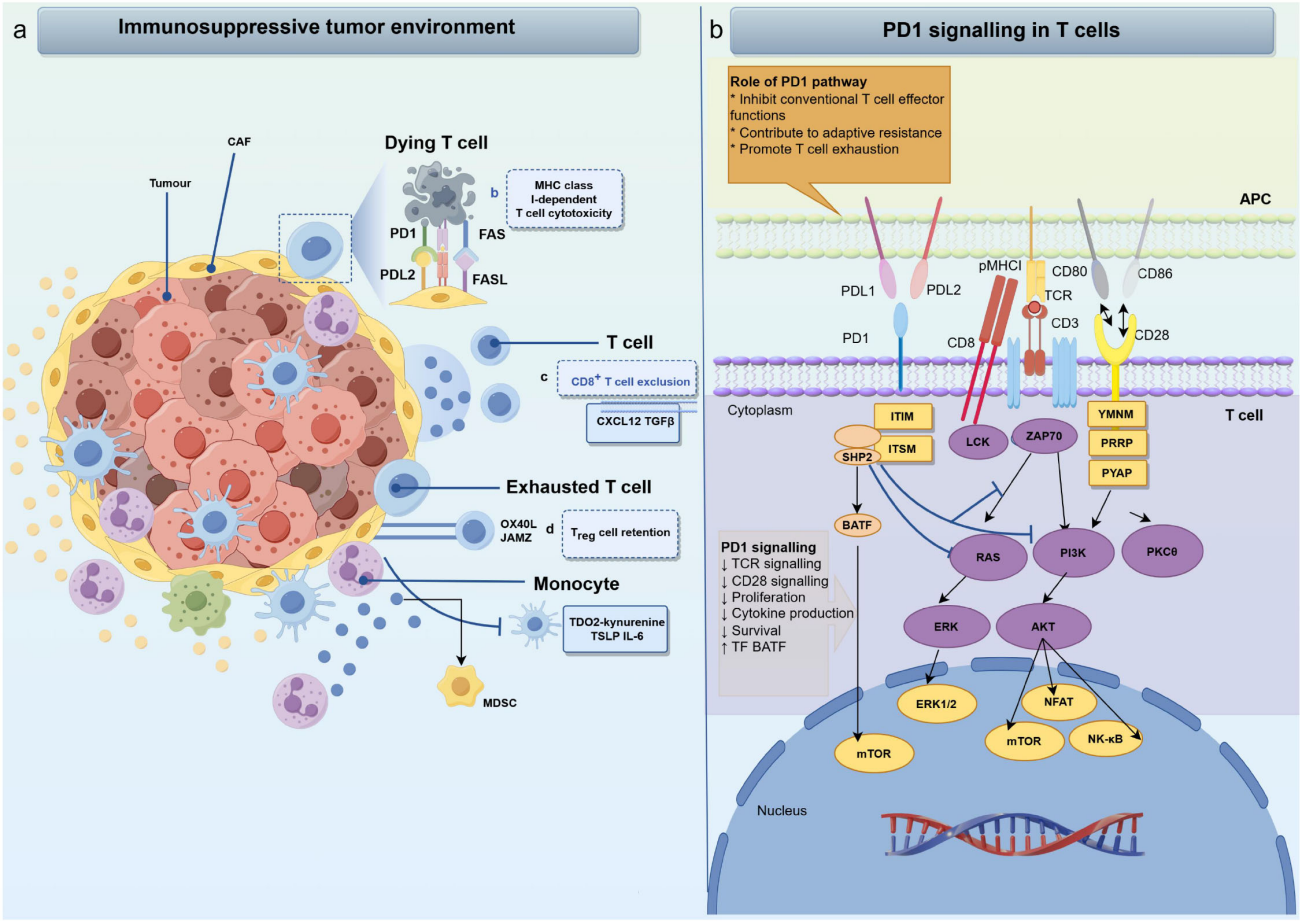
Immunosuppressive Microenvironment and PD-1 Signaling Axis. **(a)** The immunosuppressive tumor microenvironment represents a dynamically orchestrated pathological ecosystem wherein malignant cells actively subvert anti-tumor immunity. This environment is characterizing by the recruitment and expansion of regulatory immune cells, sustained expression of inhibitory ligands, and secretion of soluble mediators that collectively induce T-cell dysfunction. Key mechanisms include T-cell exhaustion through chronic antigen exposure, metabolic competition, and dysregulation of co-stimulatory signaling. These pathways converge to inhibit effector T-cell activation, proliferation, and cytotoxic function while promoting immune tolerance-ultimately enabling tumor immune evasion and therapeutic resistance. **(b)** PD-1/PD-L1 binding inhibits T-cell function by recruiting SHP-2, which reduces ZAP70 phosphorylation and suppresses the RAS-MEK-ERK and PI3K-Akt pathways. This leads to decreased T-cell proliferation, activation, cytokine production, and survival.

The immunosuppressive function of PD-1 is executed through a finely tuned intracellular signaling cascade initiated upon binding to its ligands, PD-L1 or PD-L2. Engagement leads to the phosphorylation of the immune-receptor tyrosine-based inhibitory motif (ITIM) and immune-receptor tyrosine-based switch motif (ITSM) within the PD-1 cytoplasmic tail. Phosphorylated ITSM serves as the primary docking site for the Src homology region2 (SH2) domain-containing phosphatases, predominantly SHP-2, and to a lesser extent SHP-1 (23). The recruited SHP-2 phosphatase acts as the central effector, attenuating T cell activation through two primary mechanisms. The first axis involves proximal TCR signal inhibition. SHP-2 dephosphorylates key components of the T-cell receptor (TCR) signaling complex, including CD3ζ and ZAP70 (24). This directly dampens the initial activation signal. The second axis entails the suppression of distal metabolic and survival pathways. The PD-1-SHP-2 axis inhibits downstream signaling pathways critical for T cell clonal expansion, metabolic reprogramming, and survival. This includes two pathway. The PI3K-Akt-mTOR pathway, whose inhibition reduces glycolytic metabolism and promotes the nuclear retention of transcription factors like FoxO1, favoring a pro-exhaustion gene program (25–27). The RAS-MEK-ERK pathway, which is crucial for cell proliferation and differentiation (28). The collective outcome of this signal transduction is the establishment of a robust “braking” mechanism on T cells. It results in cell cycle arrest (mediated by upregulation of p27kip1), reduced cytokine production (e.g., IL-2, IFN-γ, TNF-α), impaired cytotoxic function, and the promotion of a hyporesponsive state that can progress to T cell exhaustion under conditions of chronic antigen exposure. This molecular machinery makes PD-1 a master regulator of immune activation thresholds, delivering potent inhibitory signals that are crucial for preventing excessive immune responses (29, 30). Furthermore, the PD-1/PD-L1 axis is a critical checkpoint for enforcing peripheral self-tolerance. Under homeostatic conditions, constitutive PD-L1 expression on parenchymal and antigen-presenting cells engages PD-1 on antigen-experienced T cells. This ligand-receptor interaction dampens TCR signaling via the aforementioned mechanism, effectively limiting the expansion and effector function of autoreactive T cell clones, thereby establishing and maintaining tissue-specific immunological quiescence (31–33). This core signal transduction mechanism is summarized in **Figure 1** and underpins the role of PD-1 in maintaining peripheral tolerance and its dysfunction in autoimmunity.

### 3.2 Cell-type-specific functions: a framework for context-dependent activity

The function of PD-1 is not monolithic but exhibits striking cell-type specificity, which dictates its ultimate impact on immune homeostasis or disease pathogenesis (34). This context-dependent activity stems from differential expression patterns, coupling to distinct intracellular signaling circuits, and the unique biological role of each immune subset. Understanding these nuances is critical for predicting the outcomes of PD-1 targeted interventions. The contrasting functions of PD-1 across major immune cell types involved in autoimmunity are summarized in **Table 1**. This comparative framework highlights the double-edged nature of PD-1-based interventions. For instance, augmenting PD-1 signaling may be beneficial to calm hyperactive effector T cells but could simultaneously impair the very Tregs needed for long-term tolerance. Similarly, targeting PD-1^+^ cells for depletion must consider the elimination of pathogenic γδ T cells versus the potential collateral damage to exhausted but potentially regulatable T cells (35). The following sections on specific diseases will detail how imbalances within this cell-specific framework manifest in clinical pathology (36).

**Table 1:** Cell-type-specific functions of PD-1 in autoimmunitypd-1/PD-L1 dysregulation in autoimmune diseases.

Immune cell subset	Primary role in autoimmunity	Effect of PD-1 signaling	Consequence of dysfunction & disease association
Effector T Cells (CD4^+^/CD8^+^)	Mediate tissue inflammation and damage.	Inhibitory: Delivers cell-intrinsic brake via SHP-2, suppressing TCR signaling, proliferation, and cytokine production.	Loss-of-function: Hyperactivation of autoreactive clones → SLE, RA, T1D. Chronic engagement: Can contribute to exhaustion.
Regulatory T Cells (Tregs)	Suppress immune responses, maintain tolerance.	Supportive/Modulatory: Maintains Treg stability, survival, and suppressive capacity. PD-1 signaling may enhance FoxP3 function.	Deficiency impairs Treg function: Loss of peripheral tolerance → Lupus-like autoimmunity in models.
B Cells	Produce autoantibodies, present antigen.	Regulatory: Inhibits B cell receptor signaling, dampens activation, proliferation, and antibody class-switching.	Impaired signaling: Permits expansion of autoreactive B cells (e.g., DN2 B cells in SLE) and autoantibody production.
Natural Killer (NK) Cells	Cytotoxic killing, immunoregulation.	Inhibitory: Induces an exhausted-like state, attenuating cytotoxicity and cytokine production.	Over-activation of PD-1: Contributes to NK cell exhaustion, impairing immune-regulation in RA.
γδ T Cells (e.g., Vγ4^+^)	Early source of IL-17, bridging innate/adaptive immunity.	Context-Dual: In EAE, PD-1 can limit pathogenic IL-17 production. However, PD-1^+^ Vγ4^+^ cells are themselves a pathogenic population.	Blockade may enhance pathogenicity. Targeted depletion of PD-1^+^ γδ T cells ameliorates disease.
Macrophages / Monocytes	Antigen presentation, cytokine release, tissue damage.	Cell-Extrinsic & Intrinsic: PD-1 expression on macrophages may regulate their pro-inflammatory cytokine secretion (e.g., IL-1β in RA). PD-L1 expression provides inhibitory signal to T cells.	Aberrant PD-1 on macrophages: Fuels synovial inflammation in RA.

## 4 Regulatory mechanisms of PD-1 and SNPs involved with regulation

The expression of the PD-1 gene is influenced by various regulatory mechanisms, including transcription factors, miRNAs, and DNA methylation. These mechanisms collectively regulate the expression level of the PD-1 gene, thereby affecting immune cell activation and function.

### 4.1 Transcription factors

Transcription factors are key regulators of PDCD1 expression. Transcriptional control of PDCD1 (encoding PD-1) involves synergistic and antagonistic interactions among transcription factors. Transcription factors have been found to directly or indirectly influence the transcriptional level of the PD-1 gene. Among them, FOXP3 (Forkhead Box P3) is an important transcription factor that plays a significant role in regulating T cell function and the occurrence of autoimmune diseases (37). Studies have shown that PD-L1 expression on APCs suppresses T cell immunity by engaging PD-1 in a process that contributes to the induction of regulatory T cells (Tregs). Tregs, defined by the expression of the transcription FOXP3, protect against excess inflammation and autoimmunity (38). Additionally, Nuclear Factor of Activated T cells (NFAT) binds to the PDCD1 promoter upon T-cell receptor (TCR) activation and competes with STAT5 for shared DNA binding sites, where IL-2-induced STAT5 antagonizes NFAT-mediated PD-1 upregulation (34, 39).Additionally, NF-κ B dimers can translocate to the nucleus and bind κ B motifs within PDCD1 regulatory elements to activate transcription (40). Furthermore, T cell activation triggers the PI3K-AKT-mTOR signaling pathway which upregulates Interferon Regulatory Factor 4(IRF4) (41). IRF4 then binds to enhancer regions to sustain PDCD1 transcription, thereby linking metabolic reprogramming directly to immune checkpoint expression (42, 43).

### 4.2 miRNA

MicroRNAs (miRNAs) constitute another key layer of post-transcriptional regulation for PD-1 expression (44, 45). They function primarily by binding to complementary sequences within the 3’ untranslated region (3’UTR) of target mRNAs., leading to translational repression or mRNA degradation. This mechanism allows for precise control of gene expression levels. Studies have shown that various miRNAs, such as miRNA-146a and miRNA-155, can target PD-1 mRNA, thereby modulating PD-1 expression levels and and influencing T cell function (46).For example, miR-146a directly targets the PDCD1 3’ UTR (binding site: nucleotides 1123-1129), leading to a 60-70% reduction in PD-1 protein expression in activated CD8^+^ T cells (47, 48). Notably, polymorphisms within the miR-146a binding sites are associated with increased susceptibility to systemic lupus erythematosus (SLE) (49). In SLE patients, reduced miR-146a expression (a negative regulator of NF-κ B) results in insufficient suppression of PD-1, which exacerbates inflammation (50). Consequently, miR-146a mimics have been proposed as a potential therapeutic strategy to restore PD-1 regulation and alleviate inflammation in autoimmune diseases (51). Conversely, miR-155 suppresses PD-1 in T cells but enhances PD-L1 in dendritic cells, thereby forming a negative feedback loop within the PD-1 signaling axis in autoimmunity (52, 53).

### 4.3 DNA methylation

DNA methylation is involved in PD-1 gene transcriptional control through epigenetic silencing (54, 55). DNA methylation refers to the process of adding methyl groups to DNA molecules, which affects gene transcriptional activity by altering the methylation level of DNA sequences. Research has found that CpG islands exist in the promoter region of the PD-1 gene, and their methylation level is closely related to the expression level of the PD-1 gene. When the promoter region of the PD-1 gene is methylated, it inhibits the binding of transcription factors, thereby reducing PD-1 expression levels and promoting T cell activation and function. In CD4**^+^** T cells from SLE patients, hypermethylation of the PD-1 promoter leads to insufficient PD-1 expression, resulting in impaired suppression of autoreactive T cells (56). The regulatory networks detailed above have been predominantly characterized in T cells, reflecting the primary research focus. While core principles-such as NF-κ B-mediated induction of PD-L1-are conserved across hematopoietic lineages, emerging evidence suggests cell-type-specific nuances. For instance, in B cells, lineage-defining transcription factors may co-regulate PDCD1, while in macrophages, Toll-like receptor (TLR) ligands can induce PD-L1 through pathways distinct from those in lymphocytes. A systematic, comparative elucidation of PD-1 pathway regulation across all major immune subsets remains a significant knowledge gap. Filling this gap is crucial for deciphering how cell-specific dysregulation of this checkpoint contributes to the initiation and heterogeneity of autoimmune diseases.

## 5 Role of PD-1 in immune tolerance in T cells and in other immune subsets

### 5.1 Lessons from oncology and divergent mechanisms in autoimmunity

The PD-1/PD-L1 axis exemplifies a quintessential immune checkpoint whose function exhibits a profound context-dependent duality, with opposing roles in oncology and autoimmunity. Within the tumor microenvironment (TME), tumor cells exploit this pathway to mediate immune evasion by upregulating PD-L1 expression. The binding of PD-L1 to PD-1 receptors on T cells delivers inhibitory signals that suppress T cell-mediated anti-tumor immunity (57). In malignancies such as NSCLC (58), breast cancer (59), and melanoma (60), tumor cell upregulation of PD-L1 directly drives immunosuppression. Consequently, therapeutic blockade of the PD-1/PD-L1 axis reinvigorates anti-tumor immunity, representing a cornerstone of modern cancer immunotherapy (61).

In stark contrast, within the context of autoimmunity, an intact PD-1/PD-L1 signaling axis is essential for preventing self-reactivity and maintaining immune tolerance. Genetic variations in the PDCD1 gene, such as the PD-1.3 (rs11568821) and PD-1.5 (rs2227981) polymorphisms, are associated with increased susceptibility to systemic lupus erythematosus (SLE) and rheumatoid arthritis (RA), respectively, potentially by altering the protein’s expression or function (20). Here, ligand binding triggers downstream signaling cascades that limit pathogenic lymphocyte expansion and cytokine production, safeguarding immune homeostasis (62). Dysregulation of this pathway disrupts this balance. For example, in RA, elevated PD-L2 expression correlates with disease activity, while in SLE, PD-1 expression levels demonstrate a significant positive correlation with clinical disease activity and the presence of anti-PD-1 autoantibodies (63–66). Thus, the PD-1 pathway embodies a therapeutic paradox: it is a target for blockade to rescue immunity in cancer, yet a mechanism for preservation to maintain tolerance in autoimmunity.

### 5.2 Physiological maintenance of immune tolerance across immune cells

The PD-1/PD-L1 axis is a critical regulator of peripheral immune tolerance, acting across multiple immune cell lineages beyond T cells (67). In T cells, PD-L1 expressed on antigen-presenting cells and parenchymal tissues engages PD-1 on antigen-experienced T cells. This interaction triggers the canonical inhibitory signaling cascade, attenuating TCR signals, suppressing proliferation and pro-inflammatory cytokine production (e.g., IL-2, IFN-γ, TNF-α), and thereby curtailing effector functions (68, 69). In B cells, PD-1 signaling provides a regulatory checkpoint by inhibiting B cell receptor (BCR) signaling, dampening activation, proliferation, and antibody class-switching, which helps control autoreactive B cell clones (70). In myeloid cells such as dendritic cells and macrophages, PD-L1 expression provides a vital inhibitory signal to interacting T cells. Furthermore, PD-1 expression on macrophages themselves can modulate their inflammatory cytokine secretion and phagocytic activity (10). In regulatory T cells (Tregs), PD-1 signaling supports Treg stability, survival, and suppressive capacity, thereby reinforcing their role in immune regulation (31). Thus, the PD-1/PD-L1 axis constitutes a multi−cellular checkpoint network. Its coordinated action across diverse immune cell types is indispensable for establishing and maintaining systemic immune homeostasis.

### 5.3 Dysfunction in autoimmunity: a multi-cellular perspective

Dysregulation of the PD-1 signaling pathway contributes to autoimmune pathogenesis by disrupting the coordinated multi-cellular network that maintains immune tolerance. This dysfunction manifests across several key immune cell types. First, dysregulated PD-1 signaling can lead to the concerted hyperactivation of both autoreactive T and B lymphocytes. In SLE, for instance, impaired PD-1 function is associated with the expansion of pathogenic follicular helper T (Tfh) cells and the unchecked activation of autoantibody-producing B cells (71, 72). Second, PD-1 signaling is crucial for regulatory T cell (Treg) homeostasis and function. Its deficiency, or therapeutic blockade (as seen with immune checkpoint inhibitors), can compromise Treg stability and suppressive capacity, precipitating a loss of immune tolerance and triggering or exacerbating autoimmune responses-a phenomenon observed in immune-related adverse events (irAEs) (73, 74). Third, myeloid cells can adopt a pathogenic role in settings of chronic inflammation through aberrant PD-1/PD-L1 expression. For example, in RA synovium, PD-1-positive macrophages contribute to local inflammation through enhanced release of cytokines like IL-1β. A critical consideration is the cell-type-specificity of PD-1 function, as summarized in **Table 1**. The functional outcome of PD-1 engagement is not monolithic but exhibits striking cell-type specificity. This context-dependency means that within the same disease microenvironment, PD-1 may exert inhibitory effects on effector T cells while having distinct, and sometimes paradoxically pathogenic, roles in macrophages or specific γδ T cell subsets (34). Understanding this complexity is fundamental for developing precise therapeutic strategies that target the PD-1 pathway, as interventions must account for their differential impact across the immune landscape.

## 6 Association of the PD-1 gene with autoimmune diseases

Over recent decades, the intricate biology of PD-1 has established it as a central regulator of immunity, spurring extensive research into its association with autoimmune diseases. Studies have found that PD-1 and its ligands PD-L1 and PD-L2 are generally expressed at reduced levels in immune cells such as T cells and B cells in patients with autoimmune diseases (75). A consolidated overview of these disease-specific alterations in the PD-1 pathway is provided in **Table 2**. This downregulation is thought to contribute to a breakdown in immune regulation, thereby playing a role in the pathogenesis of these conditions.

**Table 2:** PD-1/PD-L1 dysregulation in autoimmune diseases.

Disease	Involved cell type	Expression change	Genetic associations (Representative SNPs)	Function	References
SLE	CD4^+^Tfh cells, CD4^+^ T cells, B cells	Increased PD-1 expression on peripheral CD4^+^ T cells; Increased PD-L1 expression on CD19^+^ B cells in SLE patients.	PD-1.3 (rs11568821) is a well-established risk allele for SLE across multiple populations.	The PD-1/PD-L1 pathway may regulate T/B-cell hyperactivation to maintain tolerance; High PD-1 expression correlates with disease activity.	(76–78)
RA	Synovial macrophages, CD4^+^ T cells, mDCs	Increased PD-1 expression on CD4+ T cells in peripheral blood and synovium of RA patients; Increased PD-L1 expression on mDCs in synovial tissue.	PD-1.5 (rs2227981) is a replicated risk variant associated with RA susceptibility.	The PD-1/PD-L1 pathway maintains immune tolerance by inhibiting T-cell overactivation; PD-1 activation suppresses inflammation, while PD-1 deficiency exacerbates it.	(79–81)
T1D	Pancreatic CD8^+^T cells, CD4^+^T cells, Islet β-cells	Upregulated PD-L1 expression on islet β-cells (especially in inflammatory microenvironment); Decreased PD-1 expression on peripheral CD4^+^ T cells in T1D patients.	PDCD1 locus shows genetic associations, though less prominent than in SLE/RA; functional studies support its role in disease mechanisms.	PD-L1 binding to PD-1 restricts autoreactive T-cell activity, protecting β-cells from immune-mediated destruction; Low PD-1 expression correlates with disease activity.	(82–85)
EAE	Microglia (CNS)	Decreased PD-1 expression on peripheral/encephalitogenic T cells; Increased PD-L1 on CNS-resident cells (microglia/astrocytes) during peak disease	(Animal model) – Human SNP associations are inferred from mechanistic parallels rather than direct GWAS data.	Critical for limiting neuroinflammation: PD-1 KO mice develop severe EAE; PD-L1 blockade exacerbates disease; Astrocytic PD-L1 deletion increases T-cell infiltration	(86, 87)
MS	T cells, Astrocytes	Decreased PD-1 expression on peripheral T cells; Increased PD-L1 expression on astrocytes in CNS lesions of MS patients.	PDCD1 gene region has been implicated in genetic studies of MS susceptibility, with emerging evidence for shared risk with other autoimmune diseases.	The PD-1/PD-L1 pathway maintains tolerance by regulating T-cell hyper-activation; Low PD-1 expression correlates with disease activity.	(88, 89)

### 6.1 PD-1 and systemic lupus erythematosus

SLE is a chronic autoimmune disease characterized by immune system dysregulation and the production of autoantibodies, It is characterized by the immune system mistakenly attacking healthy tissues, leading to systemic inflammation and tissue damage (90).

In patients with SLE, two distinct PD-1/PD-L1-related pathological mechanisms contribute to disease progression. Downregulation of PD-L1 in renal podocytes leads to mTORC1-S6K pathway activation, resulting in enhanced CD8^+^ T cell infiltration and subsequent kidney damage; PD-1^hi^ DN2 B cells demonstrate JAK-STAT3 hyperactivation, driving excessive production of pro-inflammatory cytokines that sustain systemic inflammation (91, 92). The PD-1/PD-L1 pathway, as an immune checkpoint, exerts negative regulation of immune responses by binding to ligands and interacting with T-cell receptors (TCRs). However, in SLE, the PD-1 signaling pathway is abnormal and the body is unable to effectively negatively regulate the immune response, leading to further progression of SLE (93). The level of soluble PD-1 (sPD-1) in patients’ serum is significantly elevated, and it is positively correlated with the expression of PD-1 on the surface of T cells, indicating that sPD-1 can serve as a serological marker to assist in the diagnosis of systemic lupus erythematosus (SLE). Moreover, the expression of PD-1 on CD4^+^ T cells can help identify whether SLE patients have renal damage, providing clues for the early diagnosis of lupus nephritis (LN) (94). Studies have shown that PD-1 expression levels are often abnormal in SLE patients, which may lead to the overactivation of immune cells, particularly CD4^+^ T cells, promoting the formation of germinal centers, B cell maturation, and antibody production (76), thereby exacerbating disease progression. PD-1 is constitutively expressed in functional Tregs and supports their regulatory activity. Loss of PD-1 impairs Treg function (95). The PD-1^+^ B cell subset (e.g., double-negative B cells, DN2) is expanded in SLE patients, with enhanced secretion of IL-6 and TNF-α, promoting inflammation (96). The expansion of PD-1^+^ DN2 B cells and the altered PD-1 expression on Tregs in SLE (**Table 1**) illustrate a concerted breakdown of PD-1-mediated regulation across both adaptive arms. This multi-cellular dysfunction underscores why SLE presents with both aberrant T cell help and unchecked B cell autoantibody production. Additionally, PD-1 signaling can affect T cell metabolic reprogramming through the mTOR pathway, promoting glycolysis and exacerbating inflammation (97).

In SLE, the production of PD-1 antibodies may disrupt the immune tolerance established by PD-L1 and PD-L2 expressed on epithelial and endothelial cells (98, 99). Studies have found that PD-1-deficient mice spontaneously develop lupus-like proliferative arthritis and glomerulonephritis with IgG3 deposition (71). Further research indicates that SLE disease activity can influence the levels and proportions of PD-1 and/or PD-L1 expression in cells involved in SLE pathogenesis (100). In patients with SLE, exhausted CD8^+^ T cells exhibit high surface expression of PD-1, while activated memory B cells show significantly reduced PD-L1 expression. *In vitro* experiments demonstrate that PD-1 agonists selectively restore IFN-γ secretion in exhausted CD8^+^ T cells from IIR-SLE patients (interferon-γ-insufficient responders with systemic lupus erythematosus). Rituximab (anti-CD20 antibody) treatment eliminates PD-L1-low activated memory B cells, thereby indirectly improving CD8^+^ T cell function. Furthermore, gene therapy approaches (e.g., lentiviral transfection of PD-L1) or small-molecule drugs that enhance PD-L1 transcription can be employed to strengthen PD-1/PD-L1 binding and restore inhibitory signaling (101).

In summary, PD-1 and its ligands participate in the immune regulation of SLE by modulating the expression and activation of immune cells and parenchymal cell receptors, influencing disease onset and progression. The production of PD-1 antibodies may affect the levels and proportions of PD-1 and/or PD-L1 expression in cells, leading to the spontaneous development of SLE. Therapeutic targeting of PD-1^+^ cells with PD-1 agonist antibodies, combining PD-1 agonists with low-dose IL-2,or treatment with rituximab (an anti-CD20 monoclonal antibody), can either deplete activated memory B cells with low PD-L1 expression or upregulate PD-L1 expression. These approaches enhance immunosuppressive signaling and ameliorate SLE-mediated tissue damage. However, PD-1/PD-L1 pathway-based therapies for SLE currently remain in the investigational stage. Despite promising preclinical data, translating PD-1-axis modulation to SLE therapy faces significant hurdles. First, the heterogeneity of SLE patients-encompassing diverse autoantibody profiles and organ involvement-likely influences PD-1 pathway dysfunction and therapeutic response. Second, as outlined in **Table 1**, PD-1 has opposing effects on different lymphocyte subsets; a systemic agonist might suppress pathogenic T cells but inadvertently impair PD-1’s supportive role in Tregs. Third, the functional relevance of sPD-1 remains unclear-is it a mere biomarker, a decoy receptor, or an active player in pathogenesis? Therefore, the future promise of targeting the PD-1/PD-L1 axis in SLE is contingent upon addressing these specific challenges. Success will likely require a precision medicine approach: stratifying patients based on PD-1 pathway endotypes, developing cell-targeted or tissue-restricted therapies to navigate its dual roles, and clarifying the biology of sPD-1. Only through such focused efforts can the potential of this immune checkpoint be safely and effectively harnessed for SLE treatment.

### 6.2 PD-1 and rheumatoid arthritis

RA is a chronic autoimmune arthritis driven by a large number of inflammatory factors, primarily characterized by synovial inflammation and progressive joint destruction (102). Elevated PD-1 expression in early RA patients may reflect compensatory immune suppression, while increased co-expression of PD-1^+^ T cell exhaustion markers (e.g., TIM-3) in late-stage RA suggests functional exhaustion associated with disease progression (103). This temporal shift in PD-1 biology-from a compensatory brake to a marker of dysfunctional T cells-highlights a critical nuance in targeting this pathway. The apparent contradiction between ‘high’ and ‘low’ PD-1 expression may be reconciled by considering the quality of the PD-1 signal rather than merely its quantity. In early disease, PD-1 upregulation may represent an intact, albeit overwhelmed, feedback loop. In chronic, established RA, persistent inflammation and antigen exposure likely drive T cells into an exhausted state where PD-1 co-expression with other inhibitory receptors (e.g., TIM-3) signifies a refractory cellular phenotype rather than effective suppression. This has direct therapeutic implications: PD-1 agonism might be beneficial in early intervention to reinforce tolerance, whereas in late-stage disease, reversing exhaustion may require combination strategies that target multiple inhibitory pathways or the inflammatory microenvironment itself. While the exact pathogenesis of rheumatoid arthritis (RA) has not yet been fully elucidated, current therapeutic approaches primarily focus on managing symptoms and slowing disease progression.

Studies have shown that impaired SOCS3 expression can lead to excessive STAT1 phosphorylation and inhibition of STAT3 phosphorylation, resulting in the loss of STAT3 function, upregulation of PD-1/PD-L1, regulation of Th17 cell differentiation, and promotion of Treg cell function. Abnormal PD-1 signaling may lead to the overactivation of T cells and B cells, exacerbating joint inflammation and destruction (104). Research indicates that PD-1 expression is significantly elevated in T cells of RA patients and is associated with disease activity (105). Additionally, the percentage of Tfh-like cells is positively correlated with DAS28 and anti-CCP antibody levels, and PD-1 expression is elevated in peripheral blood B cells of RA patients, positively correlated with autoantibody levels (e.g., anti-CCP antibodies, RF), suggesting that PD-1 may participate in autoimmune responses by regulating B cell tolerance (106). Natural killer (NK) cells are essential for the pathogenesis of RA, and miRNAs may drive PD-1 expression to regulate NK cell exhaustion (107). In RA patients, PD-1-positive synovial macrophages release IL-1β through the SHP-2-Ras-MAPK signaling cascade (108, 109). This process not only enhances local inflammatory responses but also perpetuates inflammation by recruiting additional macrophages into the joints, establishing a persistent inflammatory cycle. Thus, in RA, PD-1’s role extends beyond T cells, contributing to a multi-faceted immunosuppressive microenvironment that involves NK cell exhaustion and dysregulated macrophage activation (**Table 1**). This complexity suggests that therapeutic PD-1 agonism must overcome inhibition in diverse cellular niches. Research demonstrates that Galectin-3 interacts with PD-1, counteracting its regulatory effects on both T cell and osteoclast activity (110, 111). This interaction promotes osteoclast over-activation, consequently worsening joint inflammation and accelerating bone destruction in RA patients. Furthermore, PD-1 is highly expressed in synovial macrophages, and may promote the release of inflammatory factors (e.g., TNF-α, IL-6).

In animal models, PD-1 agonists (e.g., PD-L1-Fc fusion proteins) can alleviate joint inflammation, while PD-1 inhibitors (e.g., anti-PD-1 antibodies) may exacerbate the condition, indicating the need for precise regulation of PD-1 signaling. In clinical trials, the efficacy of PD-1/PD-L1 inhibitors in treating RA is controversial, with some studies reporting the induction or exacerbation of autoimmune reactions (112). Exploring combination therapies that target PD-1 alongside other checkpoints (e.g., CTLA-4, LAG-3) is of great interest, as synergistic blockade may enhance anti−inflammatory effects (113).

PD-1 has a double-edged sword role in RA, with mechanisms involving interactions within multi-cell and multi-molecule networks. The role of PD-1 in different immune cell subsets may be opposite, and future research should combine single-cell sequencing and spatial transcriptomics to resolve the spatiotemporal heterogeneity of PD-1 signaling and develop precise immune regulation strategies. New technologies such as engineered PD-1^+^ CAR-T cells and nanoparticle-targeted delivery of PD-1 agonists are under exploration (114).

### 6.3 PD-1 and type 1 diabetes

T1D is an autoimmune disease characterized by the destruction of pancreatic β-cells and insufficient insulin secretion (115). In healthy individuals, pancreatic islet cells express PD-L1, which engages PD-1 on T cells to deliver protective inhibitory signals, thereby helping β-cells evade autoimmune destruction (116). Disease onset results from a loss of immune tolerance, driven by multiple factors including B-cell autoantibody production, expansion of autoreactive CD4^+^and CD8^+^ T cells, and innate immune system activation. If the PD-1/PD-L1 pathway is blocked in these scenarios, it will disrupt immune tolerance and ultimately lead to the development of type 1 diabetes (T1D). Recent studies have revealed that pancreatic β-cell-derived small extracellular vesicles (sEVs) express functional PD-L1 on their surface. These PD-L1^+^ sEVs can bind to PD-1 and potently inhibit CD8^+^ T cell proliferation, activation and cytotoxic function. Importantly, interferon exposure upregulates sEVs PD-L1 expression in a dose-dependent manner, suggesting its dual potential as both a predictive biomarker for type 1 diabetes progression and a therapeutic target for preventing immune-mediated β-cell destruction. This dose-dependent regulation underscores its biomarker potential. However, the precise pathophysiological roles of sEV-associated PD-L1 in dialog with neighboring islet and immune cells require further elucidation. These findings collectively highlight PD-L1^+^ sEVs as promising mediators of immunomodulation in autoimmune diabetes, warranting further investigation into their therapeutic applications (117).

Given its protective role, strategies to enhance PD-1/PD-L1 signaling have emerged as a therapeutic avenue for T1D. Non-obese diabetic (NOD) mice are a spontaneous model of T1D, sharing genetic and pathological characteristics with human disease, and are commonly used to study the cellular and molecular mechanisms underlying T1D onset. Research has shown that abnormal PD-1 signaling may lead to excessive T cell attack on pancreatic β-cells, triggering disease onset. In studies using NOD mouse models, PD-1 gene deficiency accelerates disease progression (118). Additionally, Ansari et al. found PD-L1 expression in the islets of NOD mice, which may explain why anti-PD-L1 antibodies exacerbate diabetes. CD4^+^ CD24^hi^ NKT cells regulate the stimulation of diabetogenic CD4^+^ T cells in pancreatic lymph nodes through the PD-1/PD-L1 and ICOS/ICOSL pathways, providing long-term protection against T1D (119). As an immune checkpoint molecule, PD-1 plays a regulatory role in maintaining self-tolerance and modulating autoimmunity. Generally, suppressing autoreactive lymphocyte populations is considered an effective therapeutic strategy for autoimmune diseases, including T1D. However, its clinical application has been limited due to the concurrent suppression of lymphocytes involved in normal adaptive immunity. Unlike most existing immunotherapies, therapeutic approaches targeting the PD-L1-PD-1 axis offer distinct advantages. By specifically blocking key signaling molecules to inhibit pathogenic T-cell activation while preserving normal adaptive immune function, these strategies show promising potential in improving patient outcomes. This biomarker-based approach represents a significant advancement in autoimmune disease treatment paradigms.

A study on newly hyperglycemic NOD mice revealed that genetically engineered platelets overexpressing PD-L1 (termed PD-L1-platelets) exhibited immunomodulatory effects. These PD-L1-platelets accumulated in inflamed pancreatic tissue, where they modulated autoreactive T-cell activity. Remarkably, this intervention not only protected pancreatic β-cells but also enhanced regulatory T cell (Treg) populations, thereby reinforcing pancreatic immune tolerance. Ultimately, this approach effectively maintained normoglycemia and even reversed diabetes in the newly hyperglycemic NOD mice (120).

In contrast to strategies that aim to enhance PD-1 signaling, an alternative approach focuses on the pathogenic PD-1**^+^** immune cells themselves. Emerging research demonstrates that targeted depletion of PD-1^+^ cells (or PD-1^+^ cell exhaustion) represents an effective strategy to mitigate autoimmune disease progression. The safety of this approach, particularly its impact on general immune competence, is debated. Theoretically, this approach appears unlikely to cause widespread immunodeficiency for the following reasons: First, PD-1^+^ effector cells can be replenished from PD-1-naive lymphocytes upon immune stimulation. Second, PD-1^+^ cells are predominantly localized in inflamed tissues, with negligible detection in peripheral blood and lymphoid organs. However, emerging research has revealed that CD8**^+^** T cell subsets (Tex) play regulatory roles in normal immune homeostasis. Notably, the expansion dynamics of distinct Tex subpopulations correlate with therapeutic responses to T cell-targeted treatments in T1D. The precise immunological consequences of selectively depleting PD-1**^+^** cells warrant further investigation.

In conclusion, research on targeted PD-1**^+^** cell depletion represents a pivotal area in autoimmune disease therapeutics, showing considerable promise for clinical translation.

### 6.4 PD-1 in neuroinflammatory disorders: EAE and multiple sclerosis

Experimental autoimmune encephalomyelitis (EAE) serves as a foundational model for dissecting immune-mediated demyelination in the CNS.Its translational relevance to human multiple sclerosis (MS) is highly protocol-dependent. The myelin oligodendrocyte glycoprotein (MOG) peptide-induced EAE models discussed here are now considered to best recapitulate anti-MOG antibody-associated disease (MOGAD) in mice, rather than classic MS. Nonetheless, findings from EAE provide crucial mechanistic insights into checkpoint regulation within an inflammatory CNS environment, informing broader neuroimmunology (121, 122).

In the EAE model, γ δ T cells, specifically the Vγ4^+^ subset expressing high levels of PD-1, are critical pathogenic mediators. Blocking PD-1 function with antibodies enhances the proliferation and IL-17A production of these cells, exacerbating disease. Conversely, targeted depletion of PD-1^+^ cells (including Vγ4^+^ γ δ T cells) using anti-PD-1 immunotoxins significantly attenuates EAE progression. This establishes a unique paradigm in which PD-1 serves as a marker of a pathogenic lineage, thereby justifying a therapeutic strategy of targeted depletion over signal augmentation.

Parallel investigations in patients with multiple sclerosis (MS) have identified dysregulated expression of the PD-1 pathway. A consistent finding is decreased PD-1 expression on peripheral T cells, which correlates with disease activity (89, 123). Within active CNS lesions, PD-L1 is compensatorily upregulated on resident cells, such as astrocytes and microglia. These human observations align with the fundamental neuroprotective role of the PD-1/PD-L1 axis established in EAE models (86). Together, they underscore the pathway’s potential as a therapeutic target in demyelinating diseases.

## 7 PD-1/PD-L1 Axis and polyautoimmunity

Polyautoimmunity, defined as the presence of multiple distinct autoimmune diseases in a single individual, suggests common pathogenic roots beyond organ-specific triggers. The PD-1/PD-L1 pathway emerges as a compelling candidate for such a shared mechanism, given its fundamental role in enforcing systemic immune tolerance (68).

### 7.1 Genetic and functional convergence

Polymorphisms or regulatory defects in the PDCD1 gene or its ligands could confer a shared genetic susceptibility that broadly lowers the threshold for autoimmunity. This is exemplified by specific variants, such as the PD-1.3 allele (rs11568821), which is associated with increased risk for multiple conditions including SLE, Sjögren’s syndrome, and ankylosing spondylitis (124, 125). Such pleiotropic effects could explain the familial clustering of different autoimmune diseases and the clinical observation of disease co-aggregation (e.g., SLE with RA).

### 7.2 Systemic breakdown of checkpoint enforcement

As discussed above, the PD-1/PD-L1 axis is a ubiquitous checkpoint that restrains autoreactivity in diverse tissues. A systemic functional deficit in this pathway-whether due to genetic variants, chronic inflammatory exposure, or regulatory miRNA dysregulation-could simultaneously compromise tolerance in multiple organ systems. For instance, the same alteration in PD-1 signaling that permits loss of B cell tolerance (contributing to SLE) may also impair regulation of synovial T cells (predisposing to RA) or pancreatic islet-reactive T cells (linked to T1D).

### 7.3 From single-disease to network dysregulation and therapeutic implications

The cell-type-specific functions of PD-1 (summarized in **Table 1**) provide a framework for understanding polyautoimmunity. If PD-1’s supportive role in Tregs is compromised, a global loss of immune regulation could ensue, permitting the emergence of several autoimmune conditions. Conversely, tissue-specific manifestations (e.g., lupus nephritis vs. rheumatoid synovitis) would be determined by additional local factors, such as target antigen availability, organ-specific microenvironment, and interactions with other checkpoint molecules (e.g., CTLA-4, LAG-3). This perspective reframes PD-1 not merely as a player in isolated diseases but as a potential central node in a network of autoimmune susceptibility, highlighting key future directions and therapeutic implications. It underscores the need for future research to profile PD-1 pathway status across immune cell subsets in patients with polyautoimmunity. Therapeutically, it suggests that correcting a fundamental PD-1 pathway defect (e.g., with a carefully calibrated agonist) might have broad benefits for individuals with coexisting autoimmune conditions, moving therapy toward targeting the shared “immune soil” rather than just the separate “disease seeds”.

## 8 Potential therapeutics and challenges involved

### 8.1 Therapeutic potential

Therapeutic strategies targeting the PD-1 pathway in autoimmunity leverage three complementary approaches, each offering distinct advantages over broad-spectrum immunosuppression. First, PD-1 agonists offer targeted immunomodulation distinct from broad-spectrum immune-suppressants. This strategy directly targets the core signal transduction deficit observed in many autoimmune conditions, where PD-1-mediated inhibition of effector T cells is insufficient. By pharmacologically reinforcing the PD-1/SHP-2 inhibitory axis, these agonists aim to restore the intrinsic brake on autoreactive T cell clones (126–128). For instance, Rosnilimab shows potential in restoring immune homeostasis by reducing pathogenic T cell responses (129, 130). Parallel developments include bispecific agents (e.g., GenSci120) that combine PD-1 agonism with Fc-mediated antibody-dependent cellular cytotoxicity (ADCC). This dual-mechanism not only delivers an agonistic signal but also depletes pathogenic PD-1^+^ T cells, addressing contexts where PD-1 marks, rather than inhibits, pathogenic populations. Second, combination strategies amplify efficacy through synergistic mechanisms. Recognizing that PD-1 dysfunction occurs within a broader immune dysregulation network, these approaches aim for multi-pathway correction. For example, low-dose IL-2 coadministration exploits the JAK-STAT signaling pathway to expand regulatory T cells (Tregs)-a subset whose stability and function are supported by PD-1 (**Table 1**)-thereby creating an immunosuppressive microenvironment (131, 132). Metabolic interventions further enhance this approach: since PD-1 signaling modulates T-cell glycolysis via mTORC1, adding rapamycin potentiates Th17 suppression and delays disease progression in EAE, demonstrating how targeting a shared metabolic node can synergize with checkpoint modulation (133). Third, advanced delivery systems overcome the spatial and cellular specificity limitations of systemic therapies. This is crucial given the cell-type-specific and often tissue-localized nature of PD-1’s dysfunctional roles. For example, intra-articular injection of lipid nanoparticles encapsulating PD-L1 mRNA enables localized transfection of synovial macrophages. This approach directly addresses the pro-inflammatory role of PD-1^+^ macrophages in the RA synovium by enhancing local PD-L1 expression to suppress neighboring T cells, cutting TNF-α and IL-6 by 50% in RA joints (134, 135). Similarly, PD-1-engineered CAR-Tregs represent a cell-based delivery platform that combines two principles: (1) the homing capacity of Tregs to inflamed tissues and (2) the enhancement of Treg function via PD-1 signaling (**Table 1**). These engineered cells selectively migrate to sites of autoimmunity and preserve β-cell function for more than 12 weeks in diabetic models, highlighting the transformative potential of precision targeting (136, 137).

### 8.2 Challenges and limitations

Despite its therapeutic promise, PD-1-targeted immunotherapy in autoimmunity faces three critical translational barriers. First, the biological complexity of the PD-1 pathway poses mechanistic challenges. In Tregs, PD-1 maintains suppressive function, with PD-1 deficiency leading to lupus-like nephritis (138). In contrast, in effector T cells (Teffs), chronic activation can drive them toward an exhausted state. Precise regulation is required for the same target in different cell subsets (139, 140). After PD-1 blockade, upregulation of other checkpoints such as TIM-3/LAG-3 weakens the monotherapy efficacy. Clinical data show that in RA patients unresponsive to PD-1 agonist therapy exhibit a threefold higher frequency of TIM-3^+^ T cells compared to responders, indicating compensatory upregulation of alternative checkpoints (141). A second major concern involves long-term safety liabilities. Systemic PD-1 agonism increases susceptibility to opportunistic infections. FBXO38-deficient mice (impaired PD-1 degradation) exhibit 40% higher mortality from Candida albicans infections (142–144). Neutralizing anti-PD-1 autoantibodies in 30% of SLE patients additionally compromise therapeutic efficacy through drug clearance. Translational bottlenecks further constrain clinical implementation. Current biomarkers (serum sPD-1, Tfh frequency) show limited predictive value (145, 146). Single-cell analyses reveal 10-fold intercellular heterogeneity in PD-1 signaling sensitivity among T cell subsets. This disparity between robust efficacy in murine lupus models (>80%) and modest objective response rates in human phase II trials (35–40%) underscores the limitations of preclinical models for capturing the heterogeneous human autoimmunity. A comprehensive overview of these therapeutic strategies, their mechanisms, and the associated translational hurdles is summarized in **Table 3**.

**Table 3:** PD-1/PD-L1 therapeutic strategies and translational challenges.

Therapeutic strategy		Representative agents	Core mechanism	Major challenges	References
PD-1 Agonists (Autoimmune diseases)		Rosnilimab; Peresolimab; ImmTAAI	Activates PD-1→SHP2 signaling→ Suppresses TCR pathway, reduces TNF-α/IL-17	Neutralizing autoantibodies (30% SLE patients); Treg hyporesponsiveness	(147, 148)
PD-1 Inhibitors (Cancer)		Nivolumab; Pembrolizumab; Cemiplimab	Blocks PD-1/PD-L1→Restores CD8^+^ T cell function	Compensatory TIM-3/LAG-3 upregulation; Immunosuppressive TME ("cold tumors")	(149–152)
Combination Therapies	Low-dose IL-2	Aldesleukin	STAT5 activation→ Treg expansion→ Immune tolerance	Infection risk (fungal); Dosing sensitivity	(153, 154)
	Metabolic modulation	Rapamycin	mTORC1 inhibition→ Glycolysis blockade→Th17 suppression	Myelosuppression;Systemic immunosuppression	(155, 156)
Advanced Delivery Systems	mRNA-LNP	PFHA-PEI-LNP	Synovial macrophage-targeted PD-L1 mRNA→ Local immunosuppression	Low systemic efficiency; Manufacturing scalability	(157–159)
	CAR-Tregs	BRL-203 (non-viral)	Engineered PD-1^+^ Tregs→ Homing to inflamed tissues→ Long-term suppression	Limited persistence (<6 mo); High production costs	(160)
Bifunctional Antibodies		GenSci120	PD-1 agonism + Fc-mediated ADCC→ Depletion of pathogenic T cells	NK cell-dependent efficacy; Off-target T cell depletion	(161)

## 9 Summary

The PD-1 gene plays a significant role in the study of autoimmune diseases. As a critical immune checkpoint molecule, PD-1 interacts with its ligands PD-L1 and PD-L2 to participate in the formation of immune regulatory networks. It negatively modulates T-cell activation, proliferation, and cytokine secretion, thereby maintaining immune tolerance and homeostasis. Its abnormal expression or dysfunction is closely related to the occurrence and development of various autoimmune diseases. Notably, three key mechanistic themes emerge.

First, and most fundamentally, PD-1 exhibits a profound context-dependent duality in autoimmunity. This is most starkly illustrated by its cell-type-specific functions (**Table 1**): it is protective when maintaining the stability of Tregs or limiting effector T cell attacks on pancreatic β-cells, yet pathogenic when expressed on synovial macrophages in RA or marking pathogenic Vγ4^+^ γδ T cells in EAE. This apparent contradiction can be resolved by recognizing that PD-1’s role is not intrinsically good or bad but is defined by the biological context of the cell expressing it. In a Treg, the PD-1 signal reinforces a suppressive program. In an activated macrophage within the inflammatory synovium, PD-1 may paradoxically contribute to pro-inflammatory cytokine production. Similarly, in γδ T cells, PD-1 may mark an IL-17-producing pathogenic lineage that is best eliminated rather than modulated. A major limitation of current literature is the frequent measurement of PD-1 expression in bulk tissues or peripheral blood, which obscures these critical cell-specific nuances. Future studies employing single-cell technologies are essential to dissect these opposing roles within the same disease microenvironment. Second, tissue-specific pathway failures-such as podocyte PD-L1 deficiency in lupus nephritis, β-cell PD-L1 downregulation in T1D, and synovial macrophage PD-1 hyperactivity in RA-dictate organ-specific vulnerability. Third, PD-1 dysfunction integrates with broader signaling networks, including crosstalk with PI3K-Akt/mTOR, MAPK, and JAK-STAT pathways, which collectively amplify inflammatory cascades and metabolic reprogramming. Therefore, a deeper understanding of PD-1-its structure, regulation, and disease associations-is pivotal for elucidating autoimmune pathogenesis and advancing novel diagnostic and therapeutic strategies.

Although significant progress has been made in PD-1 research, its clinical translation still faces critical challenges. The first major challenge is the cell-specific functional heterogeneity of PD-1. It exerts distinct, often opposing effects across immune cell subsets and tissue microenvironments: in immunosuppressive cells (e.g., Tregs), PD-1 enhances inhibitory activity; in pro-inflammatory cells, it can restrict activation; and in effector T cells, it balances function to prevent excessive activation or exhaustion. This complexity renders a single therapeutic strategy inadequate for navigating the intricate immune network. The second challenge is the spatiotemporal heterogeneity of PD-1 signaling. PD-1 activation and downstream signaling are dynamically regulated by cell differentiation state, tissue localization, and disease stage. The molecular basis of this spatiotemporal specificity remains incompletely understood, severely limiting precise therapeutic intervention. Mapping the expression and signaling thresholds of PD-1 at single-cell resolution across cellular subsets is therefore essential for enabling precision targeting.

To address these challenges, future research should prioritize several directions. The application of single-cell sequencing, spatial transcriptomics, and other advanced technologies is crucial to systematically map PD-1 expression profiles and signaling networks across diseases, cell subsets, and tissue microenvironments. This will clarify the molecular basis of its spatiotemporal heterogeneity and provide a foundation for precise immune modulation. Additionally, optimizing combination therapies is necessary to achieve a sustainable balance between effective immune suppression and the preservation of host anti-infection capacity.

Future progress will require a concerted effort, marked by closer integration of basic and clinical research and enhanced interdisciplinary collaboration, to fully elucidate the role of PD-1 in autoimmune diseases and translate this knowledge into effective therapies.
